# Diversity-scaling analysis of human breast milk microbiomes from population perspective

**DOI:** 10.3389/fmicb.2022.940412

**Published:** 2022-09-26

**Authors:** Hongju Chen, Bin Yi, Yuting Qiao, Kunbao Peng, Jianmei Zhang, Jinsong Li, Kun-Wen Zheng, Ping Ning, Wendy Li

**Affiliations:** ^1^College of Mathematics, Honghe University, Mengzi, China; ^2^Computational Biology and Medical Ecology Lab, State Key Laboratory of Genetic Resources and Evolution, Kunming Institute of Zoology, Chinese Academy of Sciences, Kunming, China; ^3^Kunming College of Life Sciences, University of Chinese Academy of Sciences, Kunming, China; ^4^Department of Endocrinology, Yan’an Hospital of Kunming City, Kunming, China; ^5^Physiatrics Medicine, Yan’an Hospital of Kunming City, Kunming, China; ^6^The Yunnan Red-Cross Hospital, Affiliated Hospital of Yunnan University, Kunming, China; ^7^Department of Neurology, The First People’s Hospital of Yunnan Province, Kunming, China; ^8^Chengdu Women’s and Children’s Central Hospital, School of Medicine, University of Electronic Science and Technology of China, Chengdu, China; ^9^Department of Biology, Taiyuan Normal University, Jinzhong, China

**Keywords:** human breast milk microbiome (BMM), diversity-area relationship (DAR), diversity heterogeneity, population potential diversity, ratio of individual-to-population accrual diversity (RIP)

## Abstract

Quantitative measuring the population-level diversity-scaling of human microbiomes is different from conventional approach to traditional individual-level diversity analysis, and it is of obvious significance. For example, it is well known that individuals are of significant heterogeneity with their microbiome diversities, and the population-level analysis can effectively capture such kind of individual differences. Here we reanalyze a dozen datasets of 2,115 human breast milk microbiome (BMM) samples with diversity-area relationship (DAR) to tackle the previous questions. Our focus on BMM is aimed to offer insights for supplementing the gut microbiome research from nutritional perspective. DAR is an extension to classic species-area relationship, which was discovered in the 19th century and established as one of a handful fundamental laws in community ecology. Our DAR modeling revealed the following numbers, all approximately: (i) The population-level potential diversity of BMM is 1,108 in terms of species richness (number of total species), and 67 in terms of typical species. (ii) On average, an individual carry 17% of population-level diversity in terms of species richness, and 61% in terms of typical species. (iii) The similarity (overlap) between individuals according to pair-wise diversity overlap (PDO) should be approximately 76% in terms of total species, and 92% in terms of typical species, which symbolizes the inter-individual heterogeneity. (iv) The average individual (alpha-) diversity of BMM is approximately 188 (total-species) and 37 (typical-species). (v) To deal with the potential difference among 12 BMM datasets, we conducted DAR modeling separately for each dataset, and then performed permutation tests for DAR parameters. It was found that the DAR scaling parameter that measures inter-individual heterogeneity in diversity is invariant (constant), but the population potential diversity is different among 30% of the pair-wise comparison between 12 BMM datasets. These results offer comprehensive biodiversity analyses of the BMM from host individual, inter-individual, and population level perspectives.

## Introduction

Human breast milk contains various components, including nutrients and immunologic active substances, that play a critical role in infant growth and development (Hampel et al., 2018; Wei et al., 2019; Moubareck, 2021). Breastfeeding could reduce the risk of intestinal and respiratory infections, and may protect infants from diseases such as diarrhea, inflammatory bowel disease, diabetes, otitis media and obesity (Bode, 2012; Buchanan et al., 2012; Christian et al., 2015; Lodge et al., 2016; Garcia-Larsen et al., 2018; Westerfield et al., 2018; Davisse-Paturet et al., 2019).

With the development of non-culture techniques, abundant and diverse microorganisms have been found in breast milk that was previously thought to be sterile (Hunt et al., 2011; Fitzstevens et al., 2017; Pannaraj et al., 2017; Moossavi et al., 2019). More than 820 species of human breast milk have been identified, most of which belong to Proteobacteria and Firmicutes, dominated by *Streptococcus and Staphylococcus* (Fitzstevens et al., 2017; Moossavi et al., 2019). Microbiome in mother’s breast milk can be transmitted vertically to the intestinal tract of infants and serve as an inoculation, thus influencing the development of immune and metabolic system of infant (Jost et al., 2014; Pannaraj et al., 2017; Kapourchali and Cresci, 2020; Lyons et al., 2020; Stinson et al., 2021). For example, carbohydrate, amino acid and nitrogen metabolism, as well as cobalamin synthesis, were significantly increased in stool microbiome samples from breastfed infants compared to formula-fed infants (Gueimonde et al., 2007; Zivkovic et al., 2010; Asnicar et al., 2017). In addition, some studies have suggested that the structure of microbiome in the breast environment may be associated with the risk of breast cancer and mastitis (Urbaniak et al., 2016; Tzeng et al., 2021). Urbaniak et al. (2016) found a different bacterial profile between normal adjacent tissues of women with breast cancer and tissues of healthy controls, but a similar microbial profile between normal adjacent and tumor tissues. The relative abundance of Enterobacteriaceae, Staphylococcus epidermidis and Bacillus were higher in tumor tissue of patients with breast cancer. More recently, Fu et al. (2022) demonstrated the presence of bacteria in breast cancer tumor tissue that facilitate tumor cell metastasis and colonization through specific signaling pathways. Other studies have found that the diversity of microbiome in the milk of patients with mastitis was lower than that of healthy people, and *Corynebacterium, Staphylococcus epidermidis and Staphylococcus aureus* were the main pathogens of mastitis (Angelopoulou et al., 2018).

Diversity is one of the most important ecological indicators in the study of human microbiome, and it is no exception for breast milk microbiome (BMM). However, most studies on BMM diversity have focused on the individual level, ignoring the diversity scaling across individuals at the cohort or population level. Diversity scaling at the cohort or population level investigates changes in or differences in inter-individual BMM diversity, which is equivalent to diversity heterogeneity. The heterogeneity of microbiome diversity can reveal the characteristics of the human microbiome at the population level, which may be related to the etiology and epidemiology of human microbiome related diseases. In order to effectively assess and interpret the diversity heterogeneity of microbial community, Ma (2018a,b,^2019^) proposed the diversity-area relationship (DAR) model, which is an extension of the classical species-area relationship (SAR) model by replacing species richness with diversity measures in Hill numbers. The DAR model can not only estimate diversity heterogeneity, but also obtain other derivative parameters of diversity, including pair-wise diversity overlap (PDO), maximal accrual diversity (MAD) and ratio of individual-level to population-level diversity (RIP). These parameters describe the diversity scaling at population-level from different perspectives, thus providing a useful tool for mapping biogeography. DAR model has been used to sketch out the biogeography maps of microbiomes in major human habitats (Ma, 2018a,b), as well as some specific microbial communities, including semen microbiome of men with infertility (Ma and Li, 2018), vaginal microbiome of postpartum women in rural Malawi (Li and Ma, 2019), Chinese gut microbiome across different ethnic groups and regions (Xiao et al., 2021), and human virome (Xiao and Ma, 2021). Recently, Li and Ma (2021) also used DAR to explore the influences of 23 common human microbiome-associated diseases on the microbiome diversity scaling. In the present study, we reanalyzed the dataset composed of 2,115 healthy BMM samples using the DAR model to explore the spatial scale of species diversity of healthy BMM at the cohort (population) level, which may provide a more comprehensive understanding of the human breast milk microbiome.

## Materials and methods

### Datasets of human breast milk microbiome

The BMM datasets used in this study consisted of 2,115 samples from 12 breast-milk studies. The raw sequencing data were download from NCBI’s SRA database. Kraken2 (Version-2.1.2) and Bracken (version-2.6) were used to classify the sequences according to the reference sequence database 16S_Greengenes_k2db (downloaded March 25, 2020). The brief information of datasets is listed in Supplementary Table S3. Since the purpose of this study was to explore the microbiome diversity scaling of healthy breast milk, only healthy samples from each set of data were selected for subsequent analysis.

### DAR analysis

We used Hill number (Hill, 1973; Chao et al., 2012, 2014) to measure the diversity of BMM. The Hill numbers (^*q*^*D*) is a series of diversity measures obtained by controlling the sensitivity to the relative abundance of species in the calculation by the diversity order *q*:


(1) $${}^{q}D={\left(\overset{S}{\underset{i=1}{\sum }}p_{i}^{q}\right)}^{1/\left(1-q\right)}$$

where, $p_{i}$ is the relative abundance of the species i, and S is the number of species. When *q* = 0, ^0^D is equal to species richness (S) or the number of species. When *q* = 1, ^1^D is equal to the exponential of Shannon entropy, which represents the number of common species in the microbiome. When *q* = 2, ^2^D is the reciprocal of Simpson index, which is more sensitive to the species with high abundance.

In this study, two DAR models were fitted to the BMM datasets. The first model is the power law dar (PL-DAR) model, which is defined as:


(2) $${}^{\text{q}}D=cA^{z}$$

where ^*q*^*D* is the alpha diversity measured in Hill numbers, *A* is the area, and *c* and *z* are the parameters of PL-DAR. The second DAR model is the power law with exponential cut-off (PLEC-DAR), which is defined as:


(3) $${}^{\text{q}}D=cA^{z}\exp\left(dA\right)$$

where *exp(dA)* is the exponential decay term, and *d* is the parameter with taper-off effect, which is usually negative. To facilitate the estimation of parameters, equations (2) and (3) can be transformed into the following linear form:


(4) ln(D)=ln(c)+zln(A)

(5) ln(D)=ln(c)+zln(A)+d(A)

where parameter (*z*) is the slope of PL-DAR or PLEC-DAR models after logarithmic transformation, also known as diversity scaling. The parameter *c* corresponds to the diversity of the first unit area (sample) to be accumulated in the model fitting process, so the accumulation order of area units (samples) may affect the size of parameter *c*. To address this problem, we performed 100 DAR model fits of the BMM datasets and randomly permutated the samples before each fit.

### DAR-based profiles

#### DAR profile

The DAR profile is a series of scaling parameter (*z*) of the PL-DAR or PLEC-DAR model corresponding to different diversity order (*q*).

#### PDO profile

The pair-wise diversity overlap (PDO) characterizes the similarity between two area units (samples), which is defined as:

(6) $$g=2\left(D_{A}-D_{2A}\right)/D_{A}=2-2^{z}$$

where *z* is the scaling parameter of PL-DAR. The parameter *g* usually ranges from 0 to 1. When *g* = 0, there is no overlap between the two areas, and when *g* = 1, there is complete overlap between the two areas. The PDO profile can be defined as a series of *g*-values corresponding to different diversity orders (*q*).

#### MAD profile

According to the parameters *c, z* and *d* of PLEC-DAR, we can further obtain the maximal accrual diversity (MAD or *D*_*max*_), which is calculated by the following equation:

(7) $$Max\left({}^{q}D\right)=D_{max}=\text{c}{\left(-\frac{\text{z}}{\text{d}}\right)}^{\text{z}}\exp\left(-\text{z}\right)=\text{c}A_{max}^{z}\exp\left(-\text{z}\right)$$

where $A_{max}=-z/d$ means the number of area units (samples) needed to reach the *D*_*max*_. *D*_*max*_ measures the diversity of all possible species in the microbiomes of a population or cohort, and can be viewed as the potential diversity. The MAD profile can be defined as a series of *D*_*max*_-values corresponding to different diversity orders (*q*).

#### RIP profile

The ratio of individual-level to population-level diversity (RIP) is defined as:


(8) $$RIP=c/D_{max}$$

RIP measures the extent of an individual’s microbial diversity represents the whole population since the individual species appears in the population. The RIP profile can be defined as a series of RIP-values corresponding to different diversity orders (*q*).

### Design for DAR analysis for BMM datasets

First, DAR model fitting was performed for each BMM dataset, and 24 DAR models (12 PL-DAR models and 12 PLEC-DAR models) were obtained. Then, 2,115 samples from 12 datasets were combined, and PL-DAR and PLEC-DAR models were fitted to the combined BMM dataset. In addition, we used randomization test based on 1,000 times re-sampling to test whether there is significant difference between the parameters of each pair of 12 PL-DAR or PLEC-DAR models.

## Results and discussion

### Fitting results of DAR models

In this study, we fitted the PL-DAR model and the PLEC-DAR model to the 12 BMM datasets and a dataset with all BMM samples combined. Supplementary Table S1 lists the fitting results of 12 BMM datasets. Table 1 shows the results of the both DAR models fitted to the combined dataset of 2,115 samples. The results listed in Table 1; Supplementary Table S1 include the diversity order of Hill numbers (q), DAR parameters [*z, ln(c), d, g, A*_*max*_, *D*_*max*_ and *RIP*], measures of goodness-of-fitting (*R* and *p*), and the number of successes in 100 model fittings based on resampling data (*N*).

**Table 1:** The PL-DAR and PLEC-DAR models for the BMM dataset combined all 2,115 samples.

Dataset	Diversity Order	Power Law (PL-DAR)						PL with Exponential Cut-off (PLEC-DAR)								
		*z*	*ln(c)*	*R*	*g*	*P*	*N*	*z*	*d*	*ln(c)*	*R*	*P*	*N*	*A*_*max*_	*D*_*max*_	*RIP (%)*
Combined BMM dataset	*q* = 0	0.242	5.236	0.966	0.817	0.000	100	0.329	0.000	4.831	0.986	0.000	100	2,144	1108.5	17.0
	*q* = 1	0.078	3.623	0.658	0.944	0.000	98	0.147	0.000	3.322	0.789	0.000	88	1,472	67.4	60.8
	*q* = 2	0.042	2.856	0.436	0.970	0.001	98	0.086	0.000	2.676	0.624	0.000	82	871	24.2	79.5
	*q* = 3	0.035	2.508	0.432	0.974	0.000	92	0.069	0.000	2.367	0.606	0.000	81	1,355	16.2	82.4

When *q* = 0, all PL-DAR models fitted successfully in all 100 times of re-sampling (i.e., the success rates were 100%), and the average *R* of the PL-DAR was 0.971. Compared with the PL-DAR model, the PLEC-DAR model had a relatively lower *R*-value (mean = 0.667), but its average success rate was 91%. When *q* = 1–3, the average success rate of the PL-DAR model was 90% (the average *R* = 0.984) and that of the PLEC-DAR model was 85% (the average *R* = 0.779). These results suggest that both PL-DAR and PLEC-DAR models have a good fit for healthy human BMM.

### Randomization test for the BMM datasets

**Figure 1:**
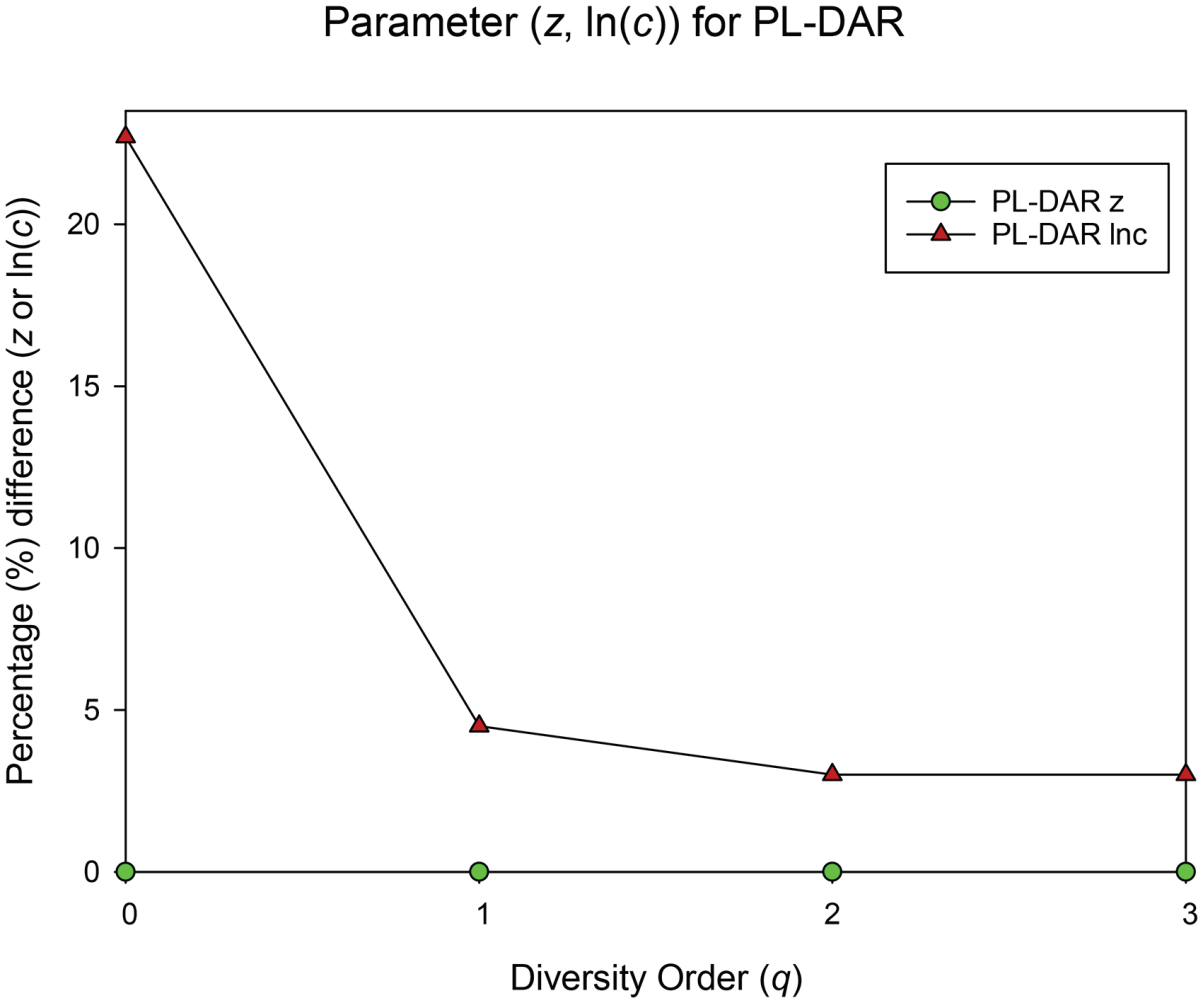
Results of randomization test: The percentage of pairwise comparisons with significant difference in the PL-DAR model parameters.

**Figure 2:**
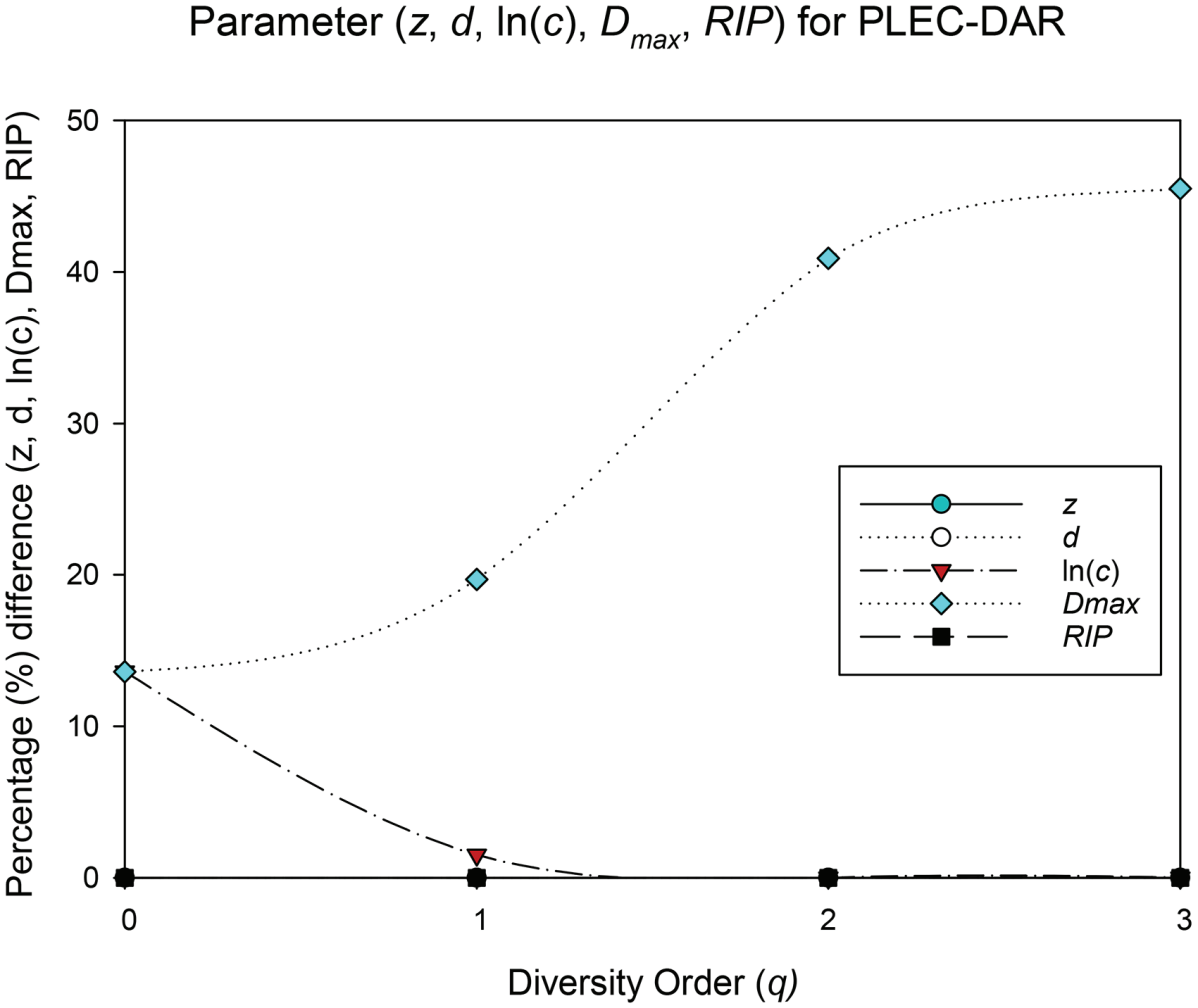
Results of randomization test: The percentage of pairwise comparisons with significant difference in the PLEC-DAR model parameters.

The randomization test was utilized to test whether there were significant differences between each pair of PL-DAR or PLEC-DAR models. Table 2 is a summary of the difference test results, the details of which are shown in Supplementary Table S2. The PL-DAR model has two parameters, *z* and ln(*c*). 8.3% (22) of the 264 pairwise comparisons had differences in ln(*c*). All pairwise comparisons showed no significant difference in parameter *z* (Figure 1). The PLEC-DAR model has three parameters, i.e., *z*, ln(*c*) and *d*. There was no significant difference in *z* and *d* between each pair of PLEC-DAR models. 3.7% (10) of the 264 pairwise comparisons had differences in ln(c) of PLEC-DAR model (Figure 2). In addition, we also tested whether there are differences between *Dmax* and *RIP* parameters between each pair-wise datasets. We found that 30% (79/264) of the comparisons were significant differences in *Dmax*, and all the comparisons were not significantly different for *RIP* (Figure 2).

**Table 2:** Summary of pairwise difference test results of 12 PL-DAR or PLEC-DAR models.

Treatment	Diversity order	PL		PLEC				
		z	ln(*c*)	z	d	ln(*c*)	*D*_*max*_	*RIP*
Percentage (%) with Significant Difference	*q* = 0	0	22.7%(15/66)	0	0	13.6%(9/66)	13.6%(9/66)	0
	*q* = 1	0	4.5%(3/66)	0	0	1.5%(1/66)	19.7%(13/66)	0
	*q* = 2	0	3.0%(2/66)	0	0	0	40.9%(27/66)	0
	*q* = 3	0	3.0%(2/66)	0	0	0	45.5%(30/66)	0

The difference test results showed that the DAR models for the 12 BMM datasets were very similar, with no significant difference in diversity scaling parameter (z) and less than 10% difference in other parameters [ln(*c*) & *d*]. It further suggested that the DAR scaling parameter that measures inter-individual heterogeneity in BMM diversity may be a constant. Combined dataset was therefore chosen to sketch out the biogeography of breast milk microbiome of healthy women.

### Biogeography map of human BMM sketched out by four profiles

**Figure 3:**
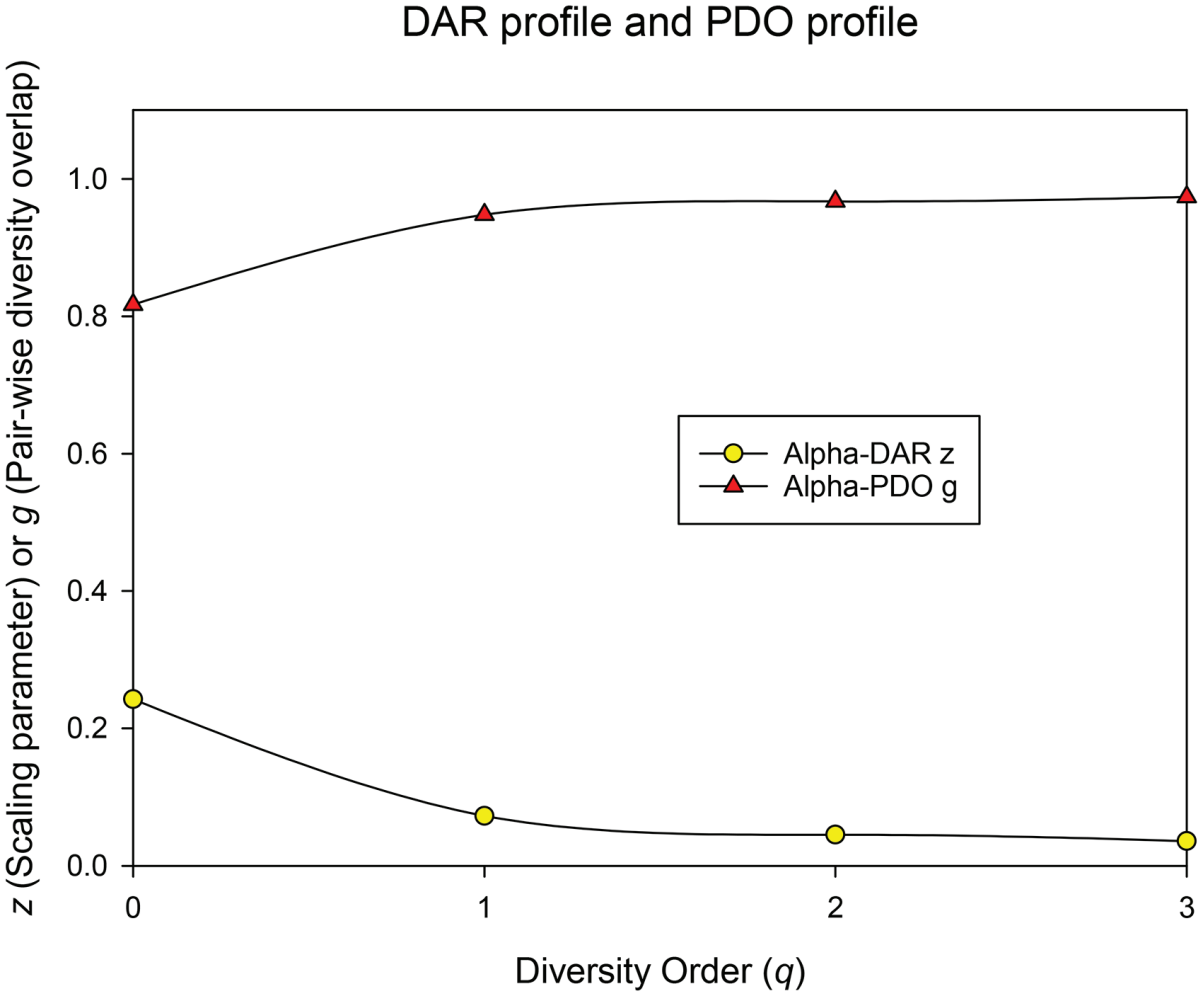
The DAR profile (*z-q*) and PDO profile (*g-q*) of the human breast milk microbiome in healthy women.

**Figure 4:**
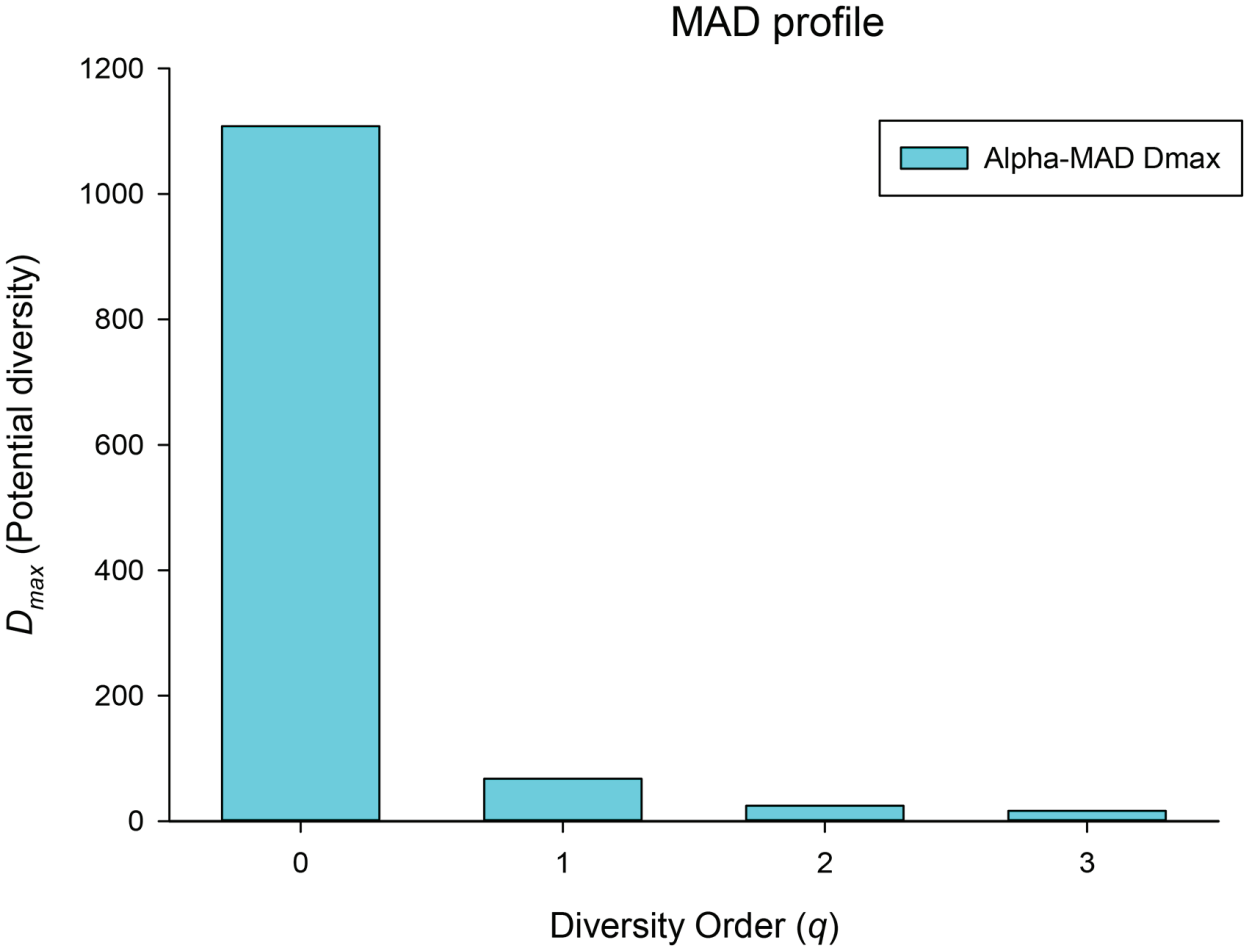
The MAD profile (D_max_-q) of the human breast milk microbiome in healthy women.

**Figure 5:**
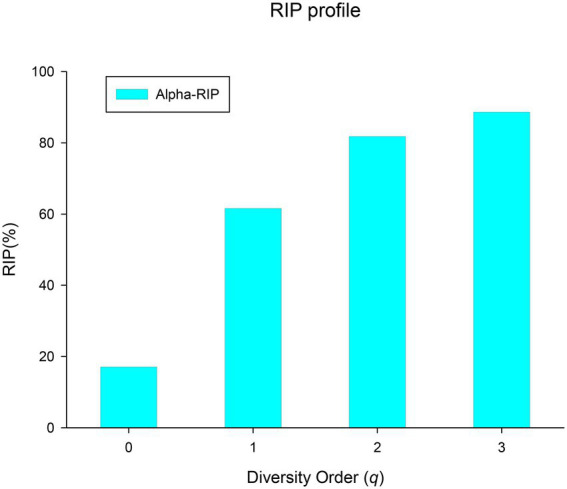
The RIP profile (RIP-q) of the human breast milk microbiome in healthy women.

(i) *DAR profile*: DAR profile, the scaling parameter (*z*) at different diversity (*q*), is *z(q)* = [0.242(0), 0.078(1), 0.042(2), 0.035(3)]. The scaling parameter *z* monotonically decreases with the diversity order (Figure 3). Diversity scaling parameter (z) characterizes the changes of BMM diversity among individuals, and is one of the most important parameters of DAR models. The higher the *z* value is, the faster the diversity of the microbiome changes among individuals in the population (or cohort). The scaling or change in BMM species richness (i.e., Hill number at *q* = 0) is the fastest, followed by the diversity of typical species and the diversity of high-abundance species (i.e., Hill numbers at *q* = 1–3). This suggests that although the number of BMM species is highly heterogeneous among individuals, the diversity of its common or core species is relatively stable.

(ii) *Pair-wise diversity overlap (PDO) profile*: The overlap parameter g can be utilized to measure the similarity between each pair of microbiomes. Parameter g and z can be viewed as two sides of the same coin, with the former estimating the similarity of microbiomes between individuals of the population (or cohort) and the latter estimating their heterogeneity. Therefore, the trend of PDO profile, which is *g(q)* = [0.817(0), 0.944(1), 0.970(2), 0.974(3)], was opposite to that of DAR, that is, it showed a monotonically increasing trend (Figure 3).

(iii) *Maximal accrual diversity (MAD) profile*: MAD or *D*_*max*_ is another important derivative parameter of the DAR model, which estimates the diversity of potential species in microbiomes of a population (or cohort). For example, *D*_*max*_ at *q* = 0 represents the number of all species that present in species pool of healthy human BMMs, and *D*_*max*_ at *q* = 1 represents the maximum number of typical (or common) species within the BMMs of a population. As shown in Figure 4; Table 1, MAD profile or *D*_*max*_ of different diversity *q* is *D*_*max*_*(q)* = [1,109(0), 67 (1), 24(2), 16(3)], which is also monotonically decreasing. It suggests that the maximum number of possible species (potential species) in the breast milk microbiome of healthy women is as high as 1,109, but there were only 67 typical (or common) species and 24 high abundance species.

(iv) *Ratio of individual-diversity to population-diversity (RIP) profile*: The derived parameter RIP measures the percentage of the microbiome diversity of a population that can be represented by the diversity of individual microbiome. As shown in equation (8) in the section of Methods, RIP is actually equal to the ratio of the average individual microbiome diversity (c) to the maximum accrual diversity (MAD or *D*_*max*_). RIP profile, that is, the RIP-value across diversity order *q*, is *RIP(q)* = [17%(0), 60.8%(1), 79.5%(2), 82.4%(3)]. MAD profile and RIP profile show opposite trends: the former decreases with the increase of *q*, while the latter increases with the increase of *q* (as shown in Figure 5). Individual microbiome can only represent 17% of the number of species in a population or cohort, but can represent 60% of the diversity of typical species and about 80% of the diversity of high-abundance species. It indicated that species with higher abundance are more important for maintaining the stability of BMM and more conserved. Similar to the results of PDO profile and DAR profile, RIP profile also reflects a high overlap or similarity among healthy women in the diversity of high-abundance species in BMM.

## Conclusion

Human breast milk not only contains nutrients essential for infant growth and development, such as human breast milk oligosaccharides (HMOS), but also has a microbiome composed of bacteria, fungi, archaea and viruses (Stinson et al., 2021). Breast milk microbiome is related to the formation and development of infant digestive system microbiome, which plays an important role in digestive metabolism, immune defense, and nervous system development of infant (Jost et al., 2014; Pannaraj et al., 2017; Hampel et al., 2018; Kapourchali and Cresci, 2020; Lyons et al., 2020; Stinson et al., 2021). Moreover, existing studies have found that breast milk microbiome is also associated with host health (Urbaniak et al., 2016; Li and Ma, 2021; Tzeng et al., 2021). Existing researches on breast milk mainly focuses on the composition of microbiome, the diversity of HMOS and the dynamic of microbiome. However, the diversity changes (scaling) of human breast milk microbiome across individuals in a population or cohort has not been explored. To fill this gap, the present study reanalyzed datasets on 2,115 breast milk microbiome collected from healthy women by using the PL-DAR and PLEC-DAR models proposed by Ma (2018a,b). Using these two DAR models, we not only estimated diversity heterogeneity of healthy breast milk microbiome, but also explored other population-level diversity attributes, including pair-wise diversity overlap (PDO), maximal accrual diversity (MAD or potential diversity), and the ratio of individual to population diversity (RIP). Based on these attributes, we have for the first time sketched out a biogeographic map of the breast milk microbiota of healthy women. These metrices may provide ecological theoretical support and tools for exploring the relationship between stability of breast milk microbiome and host health.

## Data availability statement

The original contributions presented in the study are included in the article/Supplementary material; further inquiries can be directed to the corresponding authors.

## Author contributions

HJC, BY, and YTQ conducted the data analysis. HJC and WDL wrote the manuscript. WDL revised the manuscript. HJC, PN, K-WZ, and BY conceived the study and interpreted the results. KBP, JMZ, and JSL participated the interpretation of the results and offered medical consultations. All authors contributed to the article and approved the submitted version.

## Funding

This research received funding from the following sources: The State Key Laboratory of Genetic Resources and Evolution (#GREKF21-06#, GREKF20-06, GREKF19-07); Yunnan Province Local University (Part) Basic Research for Youths (Nos. 202001BA070001-100 and 202101BA070001-018); National Science Foundation of China (grant #12161033); The Yunnan provincial department of education scientific research fund project (No. 2022 J0896); National Natural Science Foundation (NSFC) grant (No. 31970116).

## Conflict of interest

The authors declare that the research was conducted in the absence of any commercial or financial relationships that could be construed as a potential conflict of interest.

## Publisher’s note

All claims expressed in this article are solely those of the authors and do not necessarily represent those of their affiliated organizations, or those of the publisher, the editors and the reviewers. Any product that may be evaluated in this article, or claim that may be made by its manufacturer, is not guaranteed or endorsed by the publisher.
