# Supplementary material for: Diversity-scaling analysis of human breast milk microbiomes from population perspective
Source: Front Microbiol. 2022 Sep 26;13:940412. doi: 10.3389/fmicb.2022.940412 (PMC9549050; doi:10.3389/fmicb.2022.940412)
Supplement: Supplementary file 1 [file Data_Sheet_1.docx]

**Supplementary material**

**Table S1**. Fitting the two alpha- DAR (diversity-area relationship) models to the 12 groups and pooled (BMM) dataset with 100 resamples from the 2115 individuals in the human cohort

| **Dataset** | **Diversity Order** | **Power Law (PL)** | | | | | | **PL with Exponential Cutoff (PLEC)** | | | | | | | | |
| --- | --- | --- | --- | --- | --- | --- | --- | --- | --- | --- | --- | --- | --- | --- | --- | --- |
|  |  | ***z*** | **ln(*c*)** | ***R*** | ***g*** | ***P*-value** | ***N*** | ***z*** | ***d*** | **ln(*c*)** | ***R*** | ***P*-value** | ***N*** | ***Amax*** | ***Dmax*** | ***RIP (%)*** |
| Dataset#1 | *q* = 0 | 0.437 | 4.662 | 0.952 | 0.642 | 0.000 | 100 | 0.641 | -0.014 | 4.408 | 0.970 | 0.000 | 85 | 104.001 | 571.546 | 20.703 |
|  | *q* = 1 | 0.024 | 2.909 | 0.575 | 0.962 | 0.005 | 47 | 0.425 | -0.032 | 2.561 | 0.716 | 0.002 | 80 | 13.477 | 29.233 | 99.497 |
|  | *q* = 2 | -0.128 | 2.362 | 0.541 | 1.069 | 0.006 | 61 | 0.350 | -0.033 | 1.823 | 0.706 | 0.003 | 82 | 13.932 | 11.642 | 120.460 |
|  | *q* = 3 | -0.145 | 2.154 | 0.544 | 1.083 | 0.006 | 62 | 0.317 | -0.030 | 1.577 | 0.702 | 0.002 | 82 | 80.330 | 8.386 | 126.551 |
| Dataset#2 | *q* = 0 | 0.552 | 3.365 | 0.970 | 0.527 | 0.000 | 100 | 0.822 | -0.034 | 3.148 | 0.979 | 0.000 | 66 | 40.542 | 163.388 | 18.277 |
|  | *q* = 1 | 0.369 | 2.732 | 0.868 | 0.701 | 0.001 | 98 | 0.629 | -0.039 | 2.625 | 0.918 | 0.000 | 66 | 23.016 | 44.988 | 41.063 |
|  | *q* = 2 | 0.336 | 2.108 | 0.744 | 0.730 | 0.005 | 64 | 0.516 | -0.038 | 2.211 | 0.813 | 0.004 | 65 | 13.656 | 22.182 | 57.527 |
|  | *q* = 3 | 0.295 | 1.897 | 0.714 | 0.763 | 0.007 | 54 | 0.534 | -0.050 | 2.013 | 0.802 | 0.005 | 57 | 12.346 | 17.300 | 63.255 |
| Dataset#3 | *q* = 0 | 0.319 | 4.899 | 0.975 | 0.753 | 0.000 | 100 | 0.416 | -0.002 | 4.645 | 0.988 | 0.000 | 100 | 300.214 | 718.962 | 18.841 |
|  | *q* = 1 | 0.127 | 3.028 | 0.737 | 0.907 | 0.000 | 97 | 0.237 | -0.002 | 2.755 | 0.822 | 0.001 | 96 | 191.398 | 40.376 | 55.471 |
|  | *q* = 2 | 0.073 | 2.211 | 0.606 | 0.946 | 0.000 | 89 | 0.165 | -0.001 | 1.986 | 0.712 | 0.000 | 84 | 179.852 | 13.575 | 74.506 |
|  | *q* = 3 | 0.052 | 1.928 | 0.550 | 0.962 | 0.001 | 96 | 0.129 | -0.001 | 1.727 | 0.682 | 0.000 | 87 | 213.552 | 9.198 | 79.751 |
| Dataset#4 | *q* = 0 | 0.426 | 3.582 | 0.970 | 0.655 | 0.000 | 100 | 0.559 | -0.003 | 3.289 | 0.982 | 0.000 | 91 | 252.307 | 291.344 | 13.042 |
|  | *q* = 1 | 0.232 | 2.832 | 0.839 | 0.824 | 0.000 | 100 | 0.408 | -0.004 | 2.450 | 0.918 | 0.000 | 95 | 138.402 | 50.961 | 35.697 |
|  | *q* = 2 | 0.194 | 2.337 | 0.753 | 0.854 | 0.000 | 97 | 0.354 | -0.004 | 2.015 | 0.853 | 0.000 | 93 | 108.235 | 26.046 | 44.981 |
|  | *q* = 3 | 0.183 | 2.090 | 0.723 | 0.863 | 0.000 | 97 | 0.325 | -0.004 | 1.814 | 0.819 | 0.000 | 91 | 154.926 | 19.605 | 47.693 |
| Dataset#5 | *q* = 0 | 0.410 | 4.268 | 0.980 | 0.670 | 0.000 | 100 | 0.531 | -0.005 | 4.065 | 0.990 | 0.000 | 95 | 195.852 | 431.792 | 17.132 |
|  | *q* = 1 | 0.206 | 3.055 | 0.763 | 0.843 | 0.001 | 97 | 0.371 | -0.008 | 2.803 | 0.859 | 0.000 | 92 | 71.311 | 50.250 | 48.414 |
|  | *q* = 2 | 0.178 | 2.287 | 0.664 | 0.863 | 0.002 | 88 | 0.295 | -0.006 | 2.156 | 0.742 | 0.001 | 84 | 77.044 | 21.220 | 60.259 |
|  | *q* = 3 | 0.170 | 1.952 | 0.663 | 0.869 | 0.002 | 81 | 0.255 | -0.005 | 1.913 | 0.727 | 0.001 | 77 | 60.306 | 14.795 | 66.629 |
| Dataset#6 | *q* = 0 | 0.355 | 4.162 | 0.979 | 0.721 | 0.000 | 100 | 0.438 | -0.001 | 3.903 | 0.987 | 0.000 | 94 | 884.851 | 549.393 | 12.103 |
|  | *q* = 1 | 0.104 | 2.374 | 0.702 | 0.924 | 0.000 | 98 | 0.196 | -0.001 | 2.091 | 0.800 | 0.000 | 87 | 312.967 | 19.793 | 57.346 |
|  | *q* = 2 | 0.032 | 1.696 | 0.574 | 0.976 | 0.001 | 91 | 0.082 | 0.000 | 1.540 | 0.665 | 0.000 | 80 | 360.921 | 6.680 | 86.261 |
|  | *q* = 3 | 0.018 | 1.460 | 0.560 | 0.987 | 0.001 | 91 | 0.053 | 0.000 | 1.343 | 0.637 | 0.001 | 77 | 290.078 | 4.816 | 92.636 |
| Dataset#7 | *q* = 0 | 0.427 | 4.278 | 0.965 | 0.654 | 0.000 | 100 | 0.605 | -0.011 | 4.035 | 0.982 | 0.000 | 91 | 438.472 | 426.019 | 19.577 |
|  | *q* = 1 | 0.268 | 2.902 | 0.841 | 0.793 | 0.000 | 100 | 0.508 | -0.015 | 2.583 | 0.931 | 0.000 | 90 | 749.838 | 52.146 | 38.902 |
|  | *q* = 2 | 0.239 | 2.377 | 0.800 | 0.816 | 0.000 | 97 | 0.469 | -0.015 | 2.098 | 0.898 | 0.000 | 89 | 51.679 | 26.796 | 44.521 |
|  | *q* = 3 | 0.234 | 2.152 | 0.789 | 0.821 | 0.000 | 97 | 0.458 | -0.015 | 1.882 | 0.891 | 0.000 | 87 | 85.423 | 21.690 | 44.468 |
| Dataset#8 | *q* = 0 | 0.482 | 3.512 | 0.964 | 0.600 | 0.000 | 100 | 0.671 | -0.007 | 3.181 | 0.978 | 0.000 | 87 | 213.785 | 297.359 | 12.932 |
|  | *q* = 1 | 0.298 | 2.490 | 0.871 | 0.768 | 0.000 | 100 | 0.506 | -0.008 | 2.131 | 0.927 | 0.000 | 89 | 78.114 | 42.141 | 30.966 |
|  | *q* = 2 | 0.244 | 2.023 | 0.795 | 0.813 | 0.000 | 100 | 0.429 | -0.008 | 1.714 | 0.869 | 0.000 | 85 | 79.764 | 21.361 | 39.447 |
|  | *q* = 3 | 0.230 | 1.799 | 0.775 | 0.824 | 0.000 | 99 | 0.392 | -0.007 | 1.539 | 0.842 | 0.000 | 84 | 147.736 | 16.834 | 41.727 |
| Dataset#9 | *q* = 0 | 0.353 | 4.295 | 0.982 | 0.722 | 0.000 | 100 | 0.437 | -0.002 | 4.096 | 0.990 | 0.000 | 90 | 474.722 | 488.861 | 15.642 |
|  | *q* = 1 | 0.191 | 2.360 | 0.764 | 0.856 | 0.000 | 98 | 0.275 | -0.002 | 2.197 | 0.849 | 0.000 | 79 | 211.050 | 28.763 | 46.604 |
|  | *q* = 2 | 0.081 | 1.615 | 0.528 | 0.938 | 0.003 | 88 | 0.110 | -0.001 | 1.610 | 0.664 | 0.000 | 82 | 82.662 | 7.770 | 82.657 |
|  | *q* = 3 | 0.039 | 1.399 | 0.522 | 0.969 | 0.002 | 83 | 0.059 | -0.001 | 1.412 | 0.652 | 0.000 | 81 | 288.430 | 5.122 | 104.408 |
| Dataset#10 | *q* = 0 | 0.301 | 4.486 | 0.987 | 0.768 | 0.000 | 100 | 0.358 | -0.001 | 4.304 | 0.993 | 0.000 | 99 | 994.266 | 565.392 | 16.034 |
|  | *q* = 1 | 0.087 | 3.392 | 0.801 | 0.937 | 0.000 | 100 | 0.161 | -0.001 | 3.161 | 0.901 | 0.000 | 98 | 356.222 | 49.212 | 61.078 |
|  | *q* = 2 | 0.071 | 2.939 | 0.675 | 0.949 | 0.000 | 100 | 0.153 | -0.001 | 2.683 | 0.830 | 0.000 | 99 | 300.659 | 28.734 | 66.844 |
|  | *q* = 3 | 0.073 | 2.710 | 0.654 | 0.948 | 0.000 | 99 | 0.155 | -0.001 | 2.451 | 0.803 | 0.000 | 99 | 281.521 | 23.007 | 66.976 |
| Dataset#11 | *q* = 0 | 0.309 | 4.859 | 0.975 | 0.761 | 0.000 | 100 | 0.395 | -0.001 | 4.602 | 0.987 | 0.000 | 100 | 2446.589 | 791.696 | 16.898 |
|  | *q* = 1 | 0.121 | 3.169 | 0.779 | 0.912 | 0.000 | 98 | 0.230 | -0.001 | 2.842 | 0.873 | 0.000 | 96 | 226.970 | 46.644 | 52.695 |
|  | *q* = 2 | 0.101 | 2.530 | 0.679 | 0.927 | 0.000 | 97 | 0.206 | -0.001 | 2.221 | 0.801 | 0.000 | 97 | 214.553 | 22.229 | 59.425 |
|  | *q* = 3 | 0.094 | 2.280 | 0.630 | 0.932 | 0.000 | 95 | 0.197 | -0.001 | 1.986 | 0.766 | 0.000 | 94 | 226.428 | 16.847 | 62.525 |
| Dataset#12 | *q* = 0 | 0.431 | 4.188 | 0.968 | 0.649 | 0.000 | 100 | 0.605 | -0.012 | 3.963 | 0.982 | 0.000 | 86 | 148.666 | 357.028 | 19.640 |
|  | *q* = 1 | 0.222 | 2.481 | 0.685 | 0.826 | 0.003 | 87 | 0.426 | -0.016 | 2.284 | 0.786 | 0.002 | 81 | 39.394 | 27.300 | 55.577 |
|  | *q* = 2 | 0.108 | 1.857 | 0.625 | 0.914 | 0.004 | 75 | 0.292 | -0.014 | 1.652 | 0.747 | 0.002 | 75 | 37.242 | 10.094 | 76.982 |
|  | *q* = 3 | 0.051 | 1.677 | 0.619 | 0.956 | 0.003 | 76 | 0.243 | -0.013 | 1.426 | 0.742 | 0.001 | 71 | 29.630 | 6.939 | 86.782 |
| Combined | *q* = 0 | 0.242 | 5.236 | 0.966 | 0.817 | 0.000 | 100 | 0.329 | 0.000 | 4.831 | 0.986 | 0.000 | 100 | 2144 | 1108.5 | 17.0 |
|  | *q* = 1 | 0.078 | 3.623 | 0.658 | 0.944 | 0.000 | 98 | 0.147 | 0.000 | 3.322 | 0.789 | 0.000 | 88 | 1472 | 67.4 | 60.8 |
|  | *q* = 2 | 0.042 | 2.856 | 0.436 | 0.970 | 0.001 | 98 | 0.086 | 0.000 | 2.676 | 0.624 | 0.000 | 82 | 871 | 24.2 | 79.5 |
|  | *q* = 3 | 0.035 | 2.508 | 0.432 | 0.974 | 0.000 | 92 | 0.069 | 0.000 | 2.367 | 0.606 | 0.000 | 81 | 1355 | 16.2 | 82.4 |

**Table S2**. The results (percentages with significant differences) from the permutation tests for the differences in the DAR parameters with pair-wise comparisons between different datasets of healthy breast milk microbiome datasets

| **Treatment** | **Diversity order** | **PL** | | **PLEC** | | | | | |
| --- | --- | --- | --- | --- | --- | --- | --- | --- | --- |
|  |  | ***z*** | **ln(*c*)** | ***z*** | ***d*** | **ln(*c*)** | ***A_max_*** | ***D_max_*** | ***RIP*** |
| Dataset#1 *vs*. Dataset#2 | *q* = 0 | 0.693 | 0.093 | 0.769 | 0.697 | 0.177 | 0.373 | 0.050 | 0.840 |
|  | *q* = 1 | 0.596 | 0.879 | 0.816 | 0.932 | 0.958 | 0.685 | 0.584 | 0.503 |
|  | *q* = 2 | 0.452 | 0.844 | 0.836 | 0.953 | 0.733 | 0.993 | 0.373 | 0.522 |
|  | *q* = 3 | 0.438 | 0.826 | 0.772 | 0.818 | 0.682 | 0.111 | 0.280 | 0.515 |
| Dataset#1 *vs*. Dataset#3 | *q* = 0 | 0.497 | 0.654 | 0.359 | 0.313 | 0.624 | 0.561 | 0.872 | 0.793 |
|  | *q* = 1 | 0.652 | 0.864 | 0.623 | 0.128 | 0.796 | 0.288 | 0.254 | 0.208 |
|  | *q* = 2 | 0.338 | 0.843 | 0.646 | 0.135 | 0.866 | 0.228 | 0.631 | 0.318 |
|  | *q* = 3 | 0.313 | 0.755 | 0.616 | 0.146 | 0.874 | 0.323 | 0.775 | 0.317 |
| Dataset#1 *vs*. Dataset#4 | *q* = 0 | 0.967 | 0.204 | 0.856 | 0.617 | 0.260 | 0.461 | 0.200 | 0.378 |
|  | *q* = 1 | 0.771 | 0.954 | 0.967 | 0.338 | 0.922 | 0.183 | 0.466 | 0.492 |
|  | *q* = 2 | 0.619 | 0.985 | 0.997 | 0.385 | 0.861 | 0.240 | 0.254 | 0.546 |
|  | *q* = 3 | 0.566 | 0.957 | 0.993 | 0.419 | 0.832 | 0.285 | 0.175 | 0.527 |
| Dataset#1 *vs*. Dataset#5 | *q* = 0 | 0.845 | 0.437 | 0.691 | 0.504 | 0.575 | 0.399 | 0.382 | 0.660 |
|  | *q* = 1 | 0.742 | 0.888 | 0.924 | 0.513 | 0.829 | 0.300 | 0.451 | 0.528 |
|  | *q* = 2 | 0.605 | 0.948 | 0.935 | 0.513 | 0.788 | 0.241 | 0.439 | 0.531 |
|  | *q* = 3 | 0.574 | 0.851 | 0.926 | 0.522 | 0.769 | 0.602 | 0.456 | 0.519 |
| Dataset#1 *vs*. Dataset#6 | *q* = 0 | 0.757 | 0.545 | 0.566 | 0.396 | 0.523 | 0.293 | 0.991 | 0.287 |
|  | *q* = 1 | 0.850 | 0.656 | 0.607 | 0.173 | 0.645 | 0.132 | 0.391 | 0.414 |
|  | *q* = 2 | 0.627 | 0.517 | 0.534 | 0.159 | 0.760 | 0.084 | 0.196 | 0.640 |
|  | *q* = 3 | 0.566 | 0.443 | 0.514 | 0.160 | 0.776 | 0.155 | 0.171 | 0.614 |
| Dataset#1 *vs*. Dataset#7 | *q* = 0 | 0.964 | 0.481 | 0.901 | 0.847 | 0.593 | 0.115 | 0.239 | 0.908 |
|  | *q* = 1 | 0.586 | 0.993 | 0.901 | 0.771 | 0.972 | 0.011 | 0.435 | 0.386 |
|  | *q* = 2 | 0.545 | 0.984 | 0.879 | 0.823 | 0.763 | 0.308 | 0.327 | 0.436 |
|  | *q* = 3 | 0.520 | 1.000 | 0.861 | 0.817 | 0.745 | 0.891 | 0.247 | 0.401 |
| Dataset#1 *vs*. Dataset#8 | *q* = 0 | 0.854 | 0.186 | 0.956 | 0.738 | 0.270 | 0.390 | 0.124 | 0.438 |
|  | *q* = 1 | 0.631 | 0.715 | 0.900 | 0.537 | 0.687 | 0.257 | 0.649 | 0.409 |
|  | *q* = 2 | 0.559 | 0.788 | 0.911 | 0.553 | 0.920 | 0.240 | 0.478 | 0.431 |
|  | *q* = 3 | 0.524 | 0.768 | 0.915 | 0.552 | 0.968 | 0.233 | 0.395 | 0.420 |
| Dataset#1 *vs*. Dataset#9 | *q* = 0 | 0.629 | 0.494 | 0.438 | 0.344 | 0.566 | 0.369 | 0.906 | 0.587 |
|  | *q* = 1 | 0.611 | 0.595 | 0.767 | 0.213 | 0.730 | 0.144 | 0.978 | 0.321 |
|  | *q* = 2 | 0.448 | 0.457 | 0.645 | 0.217 | 0.834 | 0.417 | 0.524 | 0.549 |
|  | *q* = 3 | 0.424 | 0.387 | 0.584 | 0.214 | 0.863 | 0.128 | 0.376 | 0.703 |
| Dataset#1 *vs*. Dataset#10 | *q* = 0 | 0.346 | 0.744 | 0.163 | 0.205 | 0.798 | 0.328 | 1.000 | 0.659 |
|  | *q* = 1 | 0.851 | 0.485 | 0.401 | 0.087 | 0.346 | 0.144 | 0.105 | 0.276 |
|  | *q* = 2 | 0.535 | 0.550 | 0.603 | 0.118 | 0.331 | 0.095 | 0.096 | 0.373 |
|  | *q* = 3 | 0.546 | 0.614 | 0.686 | 0.152 | 0.368 | 0.168 | 0.103 | 0.400 |
| Dataset#1 *vs*. Dataset#11 | *q* = 0 | 0.522 | 0.760 | 0.419 | 0.350 | 0.753 | 0.058 | 0.895 | 0.575 |
|  | *q* = 1 | 0.830 | 0.832 | 0.692 | 0.154 | 0.759 | 0.157 | 0.183 | 0.329 |
|  | *q* = 2 | 0.651 | 0.907 | 0.784 | 0.150 | 0.691 | 0.106 | 0.219 | 0.441 |
|  | *q* = 3 | 0.626 | 0.917 | 0.812 | 0.179 | 0.679 | 0.195 | 0.213 | 0.468 |
| Dataset#1 *vs*. Dataset#12 | *q* = 0 | 0.976 | 0.432 | 0.915 | 0.900 | 0.551 | 0.534 | 0.152 | 0.928 |
|  | *q* = 1 | 0.700 | 0.706 | 0.999 | 0.811 | 0.803 | 0.439 | 0.943 | 0.579 |
|  | *q* = 2 | 0.690 | 0.679 | 0.937 | 0.753 | 0.886 | 0.420 | 0.871 | 0.658 |
|  | *q* = 3 | 0.715 | 0.666 | 0.911 | 0.752 | 0.880 | 0.197 | 0.848 | 0.668 |
| Dataset#2 *vs*. Dataset#3 | *q* = 0 | 0.307 | 0.012 | 0.215 | 0.282 | 0.023 | 0.529 | 0.024 | 0.951 |
|  | *q* = 1 | 0.405 | 0.721 | 0.363 | 0.428 | 0.860 | 0.302 | 0.750 | 0.669 |
|  | *q* = 2 | 0.332 | 0.902 | 0.440 | 0.501 | 0.774 | 0.250 | 0.066 | 0.721 |
|  | *q* = 3 | 0.333 | 0.962 | 0.360 | 0.359 | 0.712 | 0.203 | 0.013 | 0.731 |
| Dataset#2 *vs*. Dataset#4 | *q* = 0 | 0.644 | 0.742 | 0.502 | 0.445 | 0.813 | 0.403 | 0.699 | 0.575 |
|  | *q* = 1 | 0.695 | 0.911 | 0.626 | 0.475 | 0.795 | 0.395 | 0.824 | 0.797 |
|  | *q* = 2 | 0.680 | 0.795 | 0.760 | 0.527 | 0.797 | 0.451 | 0.775 | 0.693 |
|  | *q* = 3 | 0.746 | 0.821 | 0.684 | 0.426 | 0.794 | 0.259 | 0.843 | 0.663 |
| Dataset#2 *vs*. Dataset#5 | *q* = 0 | 0.464 | 0.088 | 0.397 | 0.385 | 0.129 | 0.203 | 0.071 | 0.907 |
|  | *q* = 1 | 0.545 | 0.681 | 0.555 | 0.520 | 0.824 | 0.464 | 0.766 | 0.796 |
|  | *q* = 2 | 0.586 | 0.851 | 0.667 | 0.602 | 0.954 | 0.295 | 0.940 | 0.954 |
|  | *q* = 3 | 0.662 | 0.965 | 0.611 | 0.500 | 0.911 | 0.367 | 0.808 | 0.957 |
| Dataset#2 *vs*. Dataset#6 | *q* = 0 | 0.499 | 0.290 | 0.334 | 0.380 | 0.253 | 0.276 | 0.211 | 0.552 |
|  | *q* = 1 | 0.335 | 0.675 | 0.341 | 0.455 | 0.491 | 0.349 | 0.009 | 0.658 |
|  | *q* = 2 | 0.192 | 0.560 | 0.349 | 0.465 | 0.344 | 0.184 | 0.003 | 0.497 |
|  | *q* = 3 | 0.183 | 0.500 | 0.279 | 0.328 | 0.318 | 0.242 | 0.003 | 0.485 |
| Dataset#2 *vs*. Dataset#7 | *q* = 0 | 0.568 | 0.109 | 0.603 | 0.563 | 0.167 | 0.066 | 0.075 | 0.913 |
|  | *q* = 1 | 0.665 | 0.784 | 0.772 | 0.560 | 0.952 | 0.029 | 0.660 | 0.926 |
|  | *q* = 2 | 0.657 | 0.662 | 0.902 | 0.595 | 0.869 | 0.425 | 0.623 | 0.625 |
|  | *q* = 3 | 0.750 | 0.678 | 0.850 | 0.446 | 0.831 | 0.238 | 0.548 | 0.514 |
| Dataset#2 *vs*. Dataset#8 | *q* = 0 | 0.765 | 0.834 | 0.741 | 0.529 | 0.966 | 0.294 | 0.422 | 0.627 |
|  | *q* = 1 | 0.775 | 0.733 | 0.767 | 0.523 | 0.500 | 0.586 | 0.901 | 0.706 |
|  | *q* = 2 | 0.711 | 0.891 | 0.856 | 0.571 | 0.497 | 0.405 | 0.926 | 0.600 |
|  | *q* = 3 | 0.785 | 0.880 | 0.769 | 0.444 | 0.499 | 0.175 | 0.951 | 0.588 |
| Dataset#2 *vs*. Dataset#9 | *q* = 0 | 0.269 | 0.039 | 0.200 | 0.277 | 0.042 | 0.281 | 0.135 | 0.839 |
|  | *q* = 1 | 0.502 | 0.667 | 0.446 | 0.471 | 0.623 | 0.318 | 0.249 | 0.907 |
|  | *q* = 2 | 0.377 | 0.585 | 0.456 | 0.547 | 0.509 | 0.497 | 0.005 | 0.637 |
|  | *q* = 3 | 0.354 | 0.563 | 0.346 | 0.419 | 0.483 | 0.103 | 0.003 | 0.449 |
| Dataset#2 *vs*. Dataset#10 | *q* = 0 | 0.082 | 0.014 | 0.071 | 0.155 | 0.017 | 0.287 | 0.122 | 0.909 |
|  | *q* = 1 | 0.196 | 0.246 | 0.201 | 0.234 | 0.257 | 0.216 | 0.859 | 0.442 |
|  | *q* = 2 | 0.306 | 0.239 | 0.310 | 0.309 | 0.379 | 0.221 | 0.351 | 0.776 |
|  | *q* = 3 | 0.407 | 0.275 | 0.325 | 0.256 | 0.442 | 0.232 | 0.347 | 0.925 |
| Dataset#2 *vs*. Dataset#11 | *q* = 0 | 0.425 | 0.041 | 0.382 | 0.413 | 0.052 | 0.028 | 0.034 | 0.872 |
|  | *q* = 1 | 0.524 | 0.671 | 0.484 | 0.458 | 0.811 | 0.411 | 0.948 | 0.784 |
|  | *q* = 2 | 0.565 | 0.686 | 0.575 | 0.487 | 0.992 | 0.352 | 0.995 | 0.967 |
|  | *q* = 3 | 0.647 | 0.718 | 0.529 | 0.360 | 0.969 | 0.306 | 0.929 | 0.980 |
| Dataset#2 *vs*. Dataset#12 | *q* = 0 | 0.582 | 0.176 | 0.627 | 0.554 | 0.270 | 0.288 | 0.109 | 0.912 |
|  | *q* = 1 | 0.640 | 0.775 | 0.727 | 0.713 | 0.744 | 0.605 | 0.209 | 0.735 |
|  | *q* = 2 | 0.483 | 0.807 | 0.732 | 0.740 | 0.588 | 0.338 | 0.078 | 0.730 |
|  | *q* = 3 | 0.449 | 0.820 | 0.636 | 0.608 | 0.554 | 0.436 | 0.042 | 0.691 |
| Dataset#3 *vs*. Dataset#4 | *q* = 0 | 0.225 | 0.003 | 0.437 | 0.525 | 0.028 | 0.842 | 0.019 | 0.306 |
|  | *q* = 1 | 0.407 | 0.748 | 0.419 | 0.447 | 0.675 | 0.645 | 0.116 | 0.453 |
|  | *q* = 2 | 0.399 | 0.854 | 0.416 | 0.484 | 0.974 | 0.562 | 0.004 | 0.465 |
|  | *q* = 3 | 0.344 | 0.817 | 0.417 | 0.471 | 0.922 | 0.633 | 0.000 | 0.455 |
| Dataset#3 *vs*. Dataset#5 | *q* = 0 | 0.369 | 0.064 | 0.477 | 0.526 | 0.145 | 0.867 | 0.073 | 0.748 |
|  | *q* = 1 | 0.564 | 0.968 | 0.540 | 0.432 | 0.938 | 0.433 | 0.169 | 0.787 |
|  | *q* = 2 | 0.482 | 0.908 | 0.594 | 0.590 | 0.788 | 0.444 | 0.026 | 0.714 |
|  | *q* = 3 | 0.414 | 0.967 | 0.611 | 0.633 | 0.789 | 0.283 | 0.020 | 0.740 |
| Dataset#3 *vs*. Dataset#6 | *q* = 0 | 0.579 | 0.032 | 0.870 | 0.590 | 0.115 | 0.113 | 0.099 | 0.140 |
|  | *q* = 1 | 0.842 | 0.282 | 0.822 | 0.677 | 0.390 | 0.504 | 0.006 | 0.958 |
|  | *q* = 2 | 0.748 | 0.427 | 0.691 | 0.647 | 0.581 | 0.397 | 0.010 | 0.742 |
|  | *q* = 3 | 0.775 | 0.452 | 0.705 | 0.680 | 0.622 | 0.716 | 0.018 | 0.733 |
| Dataset#3 *vs*. Dataset#7 | *q* = 0 | 0.518 | 0.150 | 0.381 | 0.339 | 0.155 | 0.840 | 0.148 | 0.901 |
|  | *q* = 1 | 0.444 | 0.845 | 0.359 | 0.350 | 0.792 | 0.058 | 0.175 | 0.536 |
|  | *q* = 2 | 0.329 | 0.825 | 0.340 | 0.382 | 0.875 | 0.313 | 0.004 | 0.454 |
|  | *q* = 3 | 0.277 | 0.742 | 0.278 | 0.355 | 0.835 | 0.329 | 0.001 | 0.405 |
| Dataset#3 *vs*. Dataset#8 | *q* = 0 | 0.147 | 0.002 | 0.216 | 0.300 | 0.021 | 0.869 | 0.024 | 0.299 |
|  | *q* = 1 | 0.245 | 0.391 | 0.283 | 0.369 | 0.385 | 0.440 | 0.820 | 0.375 |
|  | *q* = 2 | 0.278 | 0.812 | 0.324 | 0.455 | 0.748 | 0.472 | 0.019 | 0.383 |
|  | *q* = 3 | 0.223 | 0.876 | 0.298 | 0.462 | 0.811 | 0.614 | 0.006 | 0.373 |
| Dataset#3 *vs*. Dataset#9 | *q* = 0 | 0.536 | 0.021 | 0.840 | 0.979 | 0.159 | 0.231 | 0.030 | 0.492 |
|  | *q* = 1 | 0.547 | 0.197 | 0.832 | 0.971 | 0.397 | 0.815 | 0.078 | 0.688 |
|  | *q* = 2 | 0.949 | 0.328 | 0.801 | 0.836 | 0.621 | 0.454 | 0.071 | 0.804 |
|  | *q* = 3 | 0.914 | 0.403 | 0.736 | 0.862 | 0.679 | 0.547 | 0.084 | 0.543 |
| Dataset#3 *vs*. Dataset#10 | *q* = 0 | 0.767 | 0.130 | 0.502 | 0.283 | 0.309 | 0.119 | 0.159 | 0.536 |
|  | *q* = 1 | 0.614 | 0.392 | 0.554 | 0.438 | 0.415 | 0.368 | 0.120 | 0.784 |
|  | *q* = 2 | 0.984 | 0.228 | 0.948 | 0.674 | 0.343 | 0.564 | 0.003 | 0.843 |
|  | *q* = 3 | 0.857 | 0.243 | 0.886 | 0.830 | 0.343 | 0.745 | 0.001 | 0.772 |
| Dataset#3 *vs*. Dataset#11 | *q* = 0 | 0.859 | 0.880 | 0.805 | 0.571 | 0.902 | 0.007 | 0.487 | 0.629 |
|  | *q* = 1 | 0.952 | 0.781 | 0.965 | 0.729 | 0.890 | 0.821 | 0.305 | 0.916 |
|  | *q* = 2 | 0.799 | 0.590 | 0.833 | 0.848 | 0.741 | 0.823 | 0.019 | 0.687 |
|  | *q* = 3 | 0.734 | 0.564 | 0.722 | 0.974 | 0.727 | 0.942 | 0.008 | 0.677 |
| Dataset#3 *vs*. Dataset#12 | *q* = 0 | 0.528 | 0.100 | 0.408 | 0.367 | 0.126 | 0.815 | 0.049 | 0.890 |
|  | *q* = 1 | 0.636 | 0.414 | 0.558 | 0.404 | 0.503 | 0.344 | 0.134 | 0.997 |
|  | *q* = 2 | 0.866 | 0.621 | 0.688 | 0.535 | 0.671 | 0.314 | 0.355 | 0.950 |
|  | *q* = 3 | 0.992 | 0.703 | 0.731 | 0.526 | 0.701 | 0.210 | 0.352 | 0.850 |
| Dataset#4 *vs*. Dataset#5 | *q* = 0 | 0.901 | 0.164 | 0.912 | 0.767 | 0.246 | 0.708 | 0.164 | 0.466 |
|  | *q* = 1 | 0.892 | 0.734 | 0.892 | 0.706 | 0.608 | 0.415 | 0.963 | 0.453 |
|  | *q* = 2 | 0.936 | 0.959 | 0.822 | 0.860 | 0.850 | 0.752 | 0.554 | 0.591 |
|  | *q* = 3 | 0.949 | 0.872 | 0.825 | 0.890 | 0.906 | 0.450 | 0.513 | 0.563 |
| Dataset#4 *vs*. Dataset#6 | *q* = 0 | 0.503 | 0.174 | 0.484 | 0.428 | 0.250 | 0.303 | 0.107 | 0.854 |
|  | *q* = 1 | 0.348 | 0.456 | 0.342 | 0.367 | 0.606 | 0.551 | 0.001 | 0.468 |
|  | *q* = 2 | 0.189 | 0.288 | 0.198 | 0.302 | 0.514 | 0.290 | 0.000 | 0.330 |
|  | *q* = 3 | 0.135 | 0.259 | 0.167 | 0.304 | 0.480 | 0.536 | 0.000 | 0.299 |
| Dataset#4 *vs*. Dataset#7 | *q* = 0 | 0.998 | 0.261 | 0.876 | 0.554 | 0.282 | 0.307 | 0.332 | 0.371 |
|  | *q* = 1 | 0.846 | 0.903 | 0.731 | 0.428 | 0.843 | 0.071 | 0.929 | 0.864 |
|  | *q* = 2 | 0.800 | 0.954 | 0.716 | 0.476 | 0.916 | 0.667 | 0.918 | 0.977 |
|  | *q* = 3 | 0.764 | 0.923 | 0.708 | 0.506 | 0.934 | 0.598 | 0.759 | 0.883 |
| Dataset#4 *vs*. Dataset#8 | *q* = 0 | 0.622 | 0.869 | 0.595 | 0.465 | 0.853 | 0.842 | 0.964 | 0.987 |
|  | *q* = 1 | 0.633 | 0.544 | 0.662 | 0.543 | 0.612 | 0.535 | 0.443 | 0.759 |
|  | *q* = 2 | 0.741 | 0.630 | 0.758 | 0.658 | 0.657 | 0.774 | 0.501 | 0.789 |
|  | *q* = 3 | 0.746 | 0.666 | 0.795 | 0.727 | 0.700 | 0.948 | 0.590 | 0.818 |
| Dataset#4 *vs*. Dataset#9 | *q* = 0 | 0.338 | 0.041 | 0.430 | 0.457 | 0.091 | 0.445 | 0.106 | 0.579 |
|  | *q* = 1 | 0.787 | 0.524 | 0.630 | 0.541 | 0.761 | 0.670 | 0.040 | 0.700 |
|  | *q* = 2 | 0.530 | 0.390 | 0.475 | 0.577 | 0.678 | 0.791 | 0.002 | 0.476 |
|  | *q* = 3 | 0.391 | 0.376 | 0.394 | 0.574 | 0.674 | 0.268 | 0.000 | 0.307 |
| Dataset#4 *vs*. Dataset#10 | *q* = 0 | 0.065 | 0.003 | 0.104 | 0.201 | 0.014 | 0.374 | 0.095 | 0.578 |
|  | *q* = 1 | 0.084 | 0.112 | 0.117 | 0.192 | 0.132 | 0.313 | 0.706 | 0.144 |
|  | *q* = 2 | 0.245 | 0.170 | 0.257 | 0.289 | 0.236 | 0.287 | 0.297 | 0.353 |
|  | *q* = 3 | 0.315 | 0.199 | 0.367 | 0.380 | 0.290 | 0.554 | 0.137 | 0.444 |
| Dataset#4 *vs*. Dataset#11 | *q* = 0 | 0.243 | 0.005 | 0.401 | 0.441 | 0.031 | 0.084 | 0.036 | 0.476 |
|  | *q* = 1 | 0.395 | 0.526 | 0.402 | 0.377 | 0.586 | 0.701 | 0.409 | 0.415 |
|  | *q* = 2 | 0.484 | 0.714 | 0.494 | 0.443 | 0.785 | 0.521 | 0.169 | 0.571 |
|  | *q* = 3 | 0.496 | 0.743 | 0.569 | 0.547 | 0.810 | 0.709 | 0.276 | 0.604 |
| Dataset#4 *vs*. Dataset#12 | *q* = 0 | 0.974 | 0.354 | 0.903 | 0.600 | 0.371 | 0.663 | 0.856 | 0.366 |
|  | *q* = 1 | 0.962 | 0.660 | 0.954 | 0.590 | 0.820 | 0.396 | 0.130 | 0.480 |
|  | *q* = 2 | 0.735 | 0.608 | 0.892 | 0.737 | 0.696 | 0.370 | 0.071 | 0.507 |
|  | *q* = 3 | 0.604 | 0.660 | 0.866 | 0.747 | 0.667 | 0.191 | 0.048 | 0.438 |
| Dataset#5 *vs*. Dataset#6 | *q* = 0 | 0.732 | 0.842 | 0.689 | 0.530 | 0.790 | 0.235 | 0.905 | 0.417 |
|  | *q* = 1 | 0.602 | 0.387 | 0.546 | 0.488 | 0.381 | 0.378 | 0.003 | 0.790 |
|  | *q* = 2 | 0.374 | 0.403 | 0.457 | 0.516 | 0.442 | 0.249 | 0.000 | 0.571 |
|  | *q* = 3 | 0.308 | 0.451 | 0.439 | 0.554 | 0.434 | 0.312 | 0.000 | 0.556 |
| Dataset#5 *vs*. Dataset#7 | *q* = 0 | 0.895 | 0.983 | 0.731 | 0.507 | 0.944 | 0.177 | 0.978 | 0.724 |
|  | *q* = 1 | 0.723 | 0.798 | 0.660 | 0.559 | 0.777 | 0.037 | 0.869 | 0.618 |
|  | *q* = 2 | 0.744 | 0.896 | 0.597 | 0.553 | 0.938 | 0.633 | 0.363 | 0.539 |
|  | *q* = 3 | 0.719 | 0.766 | 0.545 | 0.552 | 0.974 | 0.681 | 0.175 | 0.413 |
| Dataset#5 *vs*. Dataset#8 | *q* = 0 | 0.518 | 0.122 | 0.567 | 0.741 | 0.193 | 0.766 | 0.144 | 0.493 |
|  | *q* = 1 | 0.546 | 0.368 | 0.649 | 0.913 | 0.384 | 0.838 | 0.385 | 0.379 |
|  | *q* = 2 | 0.716 | 0.720 | 0.671 | 0.871 | 0.607 | 0.951 | 0.977 | 0.490 |
|  | *q* = 3 | 0.747 | 0.845 | 0.670 | 0.888 | 0.659 | 0.338 | 0.620 | 0.482 |
| Dataset#5 *vs*. Dataset#9 | *q* = 0 | 0.578 | 0.936 | 0.551 | 0.484 | 0.947 | 0.247 | 0.884 | 0.779 |
|  | *q* = 1 | 0.927 | 0.376 | 0.749 | 0.513 | 0.514 | 0.459 | 0.093 | 0.949 |
|  | *q* = 2 | 0.649 | 0.461 | 0.616 | 0.664 | 0.572 | 0.963 | 0.009 | 0.671 |
|  | *q* = 3 | 0.519 | 0.513 | 0.595 | 0.704 | 0.584 | 0.144 | 0.003 | 0.500 |
| Dataset#5 *vs*. Dataset#10 | *q* = 0 | 0.324 | 0.623 | 0.204 | 0.277 | 0.475 | 0.251 | 0.829 | 0.851 |
|  | *q* = 1 | 0.358 | 0.473 | 0.273 | 0.276 | 0.426 | 0.247 | 0.877 | 0.546 |
|  | *q* = 2 | 0.487 | 0.244 | 0.512 | 0.451 | 0.371 | 0.284 | 0.031 | 0.806 |
|  | *q* = 3 | 0.538 | 0.209 | 0.658 | 0.561 | 0.388 | 0.297 | 0.027 | 0.988 |
| Dataset#5 *vs*. Dataset#11 | *q* = 0 | 0.505 | 0.234 | 0.553 | 0.543 | 0.345 | 0.031 | 0.085 | 0.960 |
|  | *q* = 1 | 0.670 | 0.871 | 0.638 | 0.503 | 0.963 | 0.492 | 0.620 | 0.882 |
|  | *q* = 2 | 0.706 | 0.722 | 0.762 | 0.621 | 0.919 | 0.506 | 0.735 | 0.980 |
|  | *q* = 3 | 0.710 | 0.623 | 0.832 | 0.679 | 0.892 | 0.389 | 0.480 | 0.895 |
| Dataset#5 *vs*. Dataset#12 | *q* = 0 | 0.856 | 0.848 | 0.749 | 0.507 | 0.826 | 0.516 | 0.491 | 0.739 |
|  | *q* = 1 | 0.941 | 0.435 | 0.893 | 0.659 | 0.558 | 0.563 | 0.096 | 0.814 |
|  | *q* = 2 | 0.766 | 0.613 | 0.997 | 0.752 | 0.594 | 0.460 | 0.117 | 0.703 |
|  | *q* = 3 | 0.613 | 0.735 | 0.978 | 0.741 | 0.599 | 0.528 | 0.138 | 0.664 |
| Dataset#6 *vs*. Dataset#7 | *q* = 0 | 0.738 | 0.847 | 0.531 | 0.391 | 0.819 | 0.585 | 0.954 | 0.297 |
|  | *q* = 1 | 0.538 | 0.567 | 0.382 | 0.389 | 0.587 | 0.189 | 0.017 | 0.517 |
|  | *q* = 2 | 0.360 | 0.454 | 0.321 | 0.424 | 0.535 | 0.209 | 0.003 | 0.363 |
|  | *q* = 3 | 0.296 | 0.404 | 0.260 | 0.424 | 0.535 | 0.366 | 0.002 | 0.293 |
| Dataset#6 *vs*. Dataset#8 | *q* = 0 | 0.379 | 0.231 | 0.295 | 0.275 | 0.236 | 0.335 | 0.272 | 0.888 |
|  | *q* = 1 | 0.276 | 0.881 | 0.251 | 0.326 | 0.963 | 0.459 | 0.007 | 0.375 |
|  | *q* = 2 | 0.154 | 0.647 | 0.181 | 0.363 | 0.827 | 0.269 | 0.000 | 0.302 |
|  | *q* = 3 | 0.129 | 0.608 | 0.164 | 0.371 | 0.782 | 0.535 | 0.001 | 0.262 |
| Dataset#6 *vs*. Dataset#9 | *q* = 0 | 0.985 | 0.696 | 0.990 | 0.626 | 0.651 | 0.423 | 0.802 | 0.427 |
|  | *q* = 1 | 0.473 | 0.985 | 0.694 | 0.718 | 0.885 | 0.726 | 0.118 | 0.648 |
|  | *q* = 2 | 0.693 | 0.881 | 0.880 | 0.856 | 0.927 | 0.319 | 0.652 | 0.941 |
|  | *q* = 3 | 0.860 | 0.916 | 0.979 | 0.867 | 0.930 | 0.990 | 0.862 | 0.791 |
| Dataset#6 *vs*. Dataset#10 | *q* = 0 | 0.208 | 0.179 | 0.330 | 0.648 | 0.213 | 0.770 | 0.781 | 0.369 |
|  | *q* = 1 | 0.763 | 0.003 | 0.771 | 0.798 | 0.040 | 0.679 | 0.000 | 0.822 |
|  | *q* = 2 | 0.607 | 0.010 | 0.635 | 0.777 | 0.081 | 0.619 | 0.000 | 0.449 |
|  | *q* = 3 | 0.558 | 0.016 | 0.543 | 0.707 | 0.115 | 0.939 | 0.000 | 0.418 |
| Dataset#6 *vs*. Dataset#11 | *q* = 0 | 0.413 | 0.025 | 0.718 | 0.866 | 0.120 | 0.091 | 0.061 | 0.268 |
|  | *q* = 1 | 0.831 | 0.059 | 0.828 | 0.772 | 0.204 | 0.472 | 0.001 | 0.807 |
|  | *q* = 2 | 0.434 | 0.082 | 0.401 | 0.539 | 0.264 | 0.384 | 0.001 | 0.320 |
|  | *q* = 3 | 0.396 | 0.102 | 0.318 | 0.458 | 0.270 | 0.680 | 0.001 | 0.328 |
| Dataset#6 *vs*. Dataset#12 | *q* = 0 | 0.724 | 0.964 | 0.555 | 0.407 | 0.918 | 0.235 | 0.858 | 0.328 |
|  | *q* = 1 | 0.616 | 0.907 | 0.521 | 0.420 | 0.819 | 0.348 | 0.293 | 0.966 |
|  | *q* = 2 | 0.705 | 0.841 | 0.515 | 0.463 | 0.892 | 0.214 | 0.129 | 0.831 |
|  | *q* = 3 | 0.833 | 0.740 | 0.521 | 0.464 | 0.912 | 0.278 | 0.163 | 0.887 |
| Dataset#7 *vs*. Dataset#8 | *q* = 0 | 0.722 | 0.200 | 0.844 | 0.779 | 0.284 | 0.220 | 0.197 | 0.416 |
|  | *q* = 1 | 0.860 | 0.532 | 0.998 | 0.628 | 0.574 | 0.046 | 0.392 | 0.657 |
|  | *q* = 2 | 0.981 | 0.582 | 0.890 | 0.590 | 0.620 | 0.616 | 0.392 | 0.799 |
|  | *q* = 3 | 0.972 | 0.577 | 0.810 | 0.583 | 0.643 | 0.383 | 0.350 | 0.900 |
| Dataset#7 *vs*. Dataset#9 | *q* = 0 | 0.643 | 0.974 | 0.398 | 0.299 | 0.886 | 0.967 | 0.947 | 0.536 |
|  | *q* = 1 | 0.752 | 0.522 | 0.519 | 0.397 | 0.681 | 0.091 | 0.096 | 0.765 |
|  | *q* = 2 | 0.539 | 0.442 | 0.394 | 0.496 | 0.634 | 0.794 | 0.020 | 0.440 |
|  | *q* = 3 | 0.443 | 0.441 | 0.353 | 0.510 | 0.633 | 0.173 | 0.006 | 0.281 |
| Dataset#7 *vs*. Dataset#10 | *q* = 0 | 0.340 | 0.694 | 0.124 | 0.157 | 0.457 | 0.518 | 0.885 | 0.652 |
|  | *q* = 1 | 0.302 | 0.413 | 0.187 | 0.223 | 0.270 | 0.165 | 0.819 | 0.347 |
|  | *q* = 2 | 0.403 | 0.383 | 0.293 | 0.303 | 0.344 | 0.273 | 0.697 | 0.478 |
|  | *q* = 3 | 0.447 | 0.405 | 0.330 | 0.336 | 0.382 | 0.446 | 0.717 | 0.495 |
| Dataset#7 *vs*. Dataset#11 | *q* = 0 | 0.552 | 0.340 | 0.453 | 0.401 | 0.368 | 0.031 | 0.201 | 0.657 |
|  | *q* = 1 | 0.644 | 0.772 | 0.480 | 0.387 | 0.777 | 0.062 | 0.679 | 0.656 |
|  | *q* = 2 | 0.668 | 0.881 | 0.533 | 0.437 | 0.885 | 0.432 | 0.348 | 0.711 |
|  | *q* = 3 | 0.644 | 0.891 | 0.531 | 0.464 | 0.904 | 0.534 | 0.202 | 0.662 |
| Dataset#7 *vs*. Dataset#12 | *q* = 0 | 0.974 | 0.849 | 0.997 | 0.948 | 0.905 | 0.111 | 0.316 | 0.993 |
|  | *q* = 1 | 0.813 | 0.566 | 0.831 | 0.962 | 0.726 | 0.023 | 0.058 | 0.499 |
|  | *q* = 2 | 0.553 | 0.495 | 0.653 | 0.940 | 0.603 | 0.637 | 0.030 | 0.334 |
|  | *q* = 3 | 0.416 | 0.540 | 0.581 | 0.930 | 0.588 | 0.249 | 0.015 | 0.283 |
| Dataset#8 *vs*. Dataset#9 | *q* = 0 | 0.163 | 0.036 | 0.177 | 0.193 | 0.067 | 0.461 | 0.186 | 0.645 |
|  | *q* = 1 | 0.546 | 0.867 | 0.435 | 0.428 | 0.952 | 0.493 | 0.211 | 0.571 |
|  | *q* = 2 | 0.432 | 0.663 | 0.382 | 0.518 | 0.901 | 0.977 | 0.011 | 0.427 |
|  | *q* = 3 | 0.326 | 0.656 | 0.339 | 0.552 | 0.879 | 0.246 | 0.000 | 0.247 |
| Dataset#8 *vs*. Dataset#10 | *q* = 0 | 0.057 | 0.012 | 0.066 | 0.106 | 0.023 | 0.377 | 0.171 | 0.637 |
|  | *q* = 1 | 0.074 | 0.041 | 0.117 | 0.202 | 0.062 | 0.290 | 0.242 | 0.146 |
|  | *q* = 2 | 0.231 | 0.083 | 0.256 | 0.322 | 0.121 | 0.309 | 0.024 | 0.298 |
|  | *q* = 3 | 0.296 | 0.100 | 0.345 | 0.419 | 0.172 | 0.710 | 0.035 | 0.362 |
| Dataset#8 *vs*. Dataset#11 | *q* = 0 | 0.225 | 0.012 | 0.273 | 0.340 | 0.038 | 0.047 | 0.036 | 0.521 |
|  | *q* = 1 | 0.330 | 0.301 | 0.340 | 0.393 | 0.324 | 0.534 | 0.526 | 0.359 |
|  | *q* = 2 | 0.448 | 0.446 | 0.442 | 0.486 | 0.471 | 0.509 | 0.798 | 0.457 |
|  | *q* = 3 | 0.459 | 0.460 | 0.497 | 0.569 | 0.520 | 0.839 | 0.995 | 0.473 |
| Dataset#8 *vs*. Dataset#12 | *q* = 0 | 0.771 | 0.281 | 0.835 | 0.770 | 0.309 | 0.552 | 0.648 | 0.379 |
|  | *q* = 1 | 0.715 | 0.991 | 0.841 | 0.701 | 0.852 | 0.590 | 0.242 | 0.371 |
|  | *q* = 2 | 0.572 | 0.851 | 0.748 | 0.803 | 0.949 | 0.451 | 0.122 | 0.367 |
|  | *q* = 3 | 0.459 | 0.889 | 0.732 | 0.792 | 0.891 | 0.155 | 0.070 | 0.315 |
| Dataset#9 *vs*. Dataset#10 | *q* = 0 | 0.268 | 0.382 | 0.325 | 0.339 | 0.417 | 0.409 | 0.710 | 0.926 |
|  | *q* = 1 | 0.199 | 0.014 | 0.419 | 0.550 | 0.075 | 0.462 | 0.003 | 0.427 |
|  | *q* = 2 | 0.924 | 0.025 | 0.797 | 0.924 | 0.120 | 0.255 | 0.001 | 0.538 |
|  | *q* = 3 | 0.765 | 0.041 | 0.628 | 1.000 | 0.155 | 0.976 | 0.002 | 0.192 |
| Dataset#9 *vs*. Dataset#11 | *q* = 0 | 0.535 | 0.089 | 0.743 | 0.697 | 0.256 | 0.065 | 0.060 | 0.769 |
|  | *q* = 1 | 0.490 | 0.103 | 0.802 | 0.820 | 0.317 | 0.954 | 0.009 | 0.747 |
|  | *q* = 2 | 0.857 | 0.139 | 0.630 | 0.963 | 0.383 | 0.460 | 0.003 | 0.481 |
|  | *q* = 3 | 0.639 | 0.191 | 0.509 | 0.919 | 0.420 | 0.682 | 0.003 | 0.311 |
| Dataset#9 *vs*. Dataset#12 | *q* = 0 | 0.540 | 0.788 | 0.393 | 0.345 | 0.748 | 0.393 | 0.816 | 0.598 |
|  | *q* = 1 | 0.899 | 0.875 | 0.686 | 0.444 | 0.909 | 0.340 | 0.897 | 0.771 |
|  | *q* = 2 | 0.918 | 0.796 | 0.664 | 0.574 | 0.968 | 0.703 | 0.617 | 0.923 |
|  | *q* = 3 | 0.964 | 0.745 | 0.648 | 0.575 | 0.980 | 0.118 | 0.587 | 0.774 |
| Dataset#10 *vs*. Dataset#11 | *q* = 0 | 0.867 | 0.153 | 0.716 | 0.548 | 0.443 | 0.086 | 0.067 | 0.862 |
|  | *q* = 1 | 0.480 | 0.411 | 0.494 | 0.477 | 0.435 | 0.199 | 0.454 | 0.536 |
|  | *q* = 2 | 0.627 | 0.230 | 0.648 | 0.608 | 0.367 | 0.336 | 0.016 | 0.709 |
|  | *q* = 3 | 0.766 | 0.299 | 0.743 | 0.657 | 0.395 | 0.569 | 0.018 | 0.832 |
| Dataset#10 *vs*. Dataset#12 | *q* = 0 | 0.271 | 0.488 | 0.115 | 0.166 | 0.297 | 0.335 | 0.777 | 0.703 |
|  | *q* = 1 | 0.457 | 0.093 | 0.304 | 0.243 | 0.129 | 0.249 | 0.012 | 0.812 |
|  | *q* = 2 | 0.872 | 0.102 | 0.609 | 0.399 | 0.151 | 0.244 | 0.010 | 0.770 |
|  | *q* = 3 | 0.921 | 0.134 | 0.748 | 0.455 | 0.161 | 0.271 | 0.009 | 0.569 |
| Dataset#11 *vs*. Dataset#12 | *q* = 0 | 0.574 | 0.291 | 0.488 | 0.454 | 0.338 | 0.024 | 0.068 | 0.690 |
|  | *q* = 1 | 0.732 | 0.421 | 0.635 | 0.450 | 0.505 | 0.440 | 0.037 | 0.932 |
|  | *q* = 2 | 0.986 | 0.421 | 0.841 | 0.574 | 0.475 | 0.409 | 0.031 | 0.644 |
|  | *q* = 3 | 0.885 | 0.466 | 0.918 | 0.610 | 0.468 | 0.308 | 0.022 | 0.525 |
|  |  | ***z*** | **ln(*c*)** | ***z*** | ***d*** | **ln(*c*)** | ***A_max_*** | ***D_max_*** | ***RIP*** |
| Percentage (%) with Significant Difference | *q* = 0 | 0 | 22.7%(15/66) | 0 | 0 | 13.6%(9/66) | 9.1%(6/66) | 13.6%(9/66) | 0 |
|  | *q* = 1 | 0 | 4.5%(3/66) | 0 | 0 | 1.5%(1/66) | 7.6%(5/66) | 19.7%(13/66) | 0 |
|  | *q* = 2 | 0 | 3.0%(2/66) | 0 | 0 | 0 | 0 | 40.9%(27/66) | 0 |
|  | *q* = 3 | 0 | 3.0%(2/66) | 0 | 0 | 0 | 0 | 45.5%(30/66) | 0 |

**Table S3**. Brief information on the microbiome datasets of breast milk samples

| **Dataset No.** | **NCBI Data Accession No.** | **Health Condition** | **Sample Source** | **Country** | **Reference** | **Sample Size** |
| --- | --- | --- | --- | --- | --- | --- |
| Dataset #1 | PRJEB34323 | Healthy | Breast milk | Indonesia | Khine WWT, et al. (2020) *Gut Microbes*. | 45 |
| Dataset #2 | PRJNA278964 | Healthy | Breast milk | USA | Hoashi M, et al. (2016) *Reprod Sci*. | 20 |
| Dataset #3 | PRJNA295847 | Healthy | Breast milk | USA | Kordy K, et al. (2020) *PLoS One*. | 241 |
| Dataset #4 | PRJNA350740 | Healthy | Breast milk | China | Li SW, et al. (2017) *Front Microbiol*. | 145 |
| Dataset #5 | PRJNA510564 | Healthy | Breast milk | South Africa | Ojo-Okunola A, et al. (2020) *Methods Protoc*. | 78 |
| Dataset #6 | PRJNA520889 | Healthy | Breast milk | South Africa | Ojo-Okunola A, et al. (2019) *Nutrients*. | 408 |
| Dataset #7 | PRJNA542027 | Healthy | Breast milk | China | - | 51 |
| Dataset #8 | PRJNA564200 | Healthy | Breast milk | Australia | - | 85 |
| Dataset #9 | PRJNA607284 | Healthy | Breast milk | Canada | Asbury MR, et al. (2020) *Cell Host Microbe*. | 204 |
| Dataset #10 | PRJNA481046 | Healthy | Breast milk | USA | Moossavi S, et al. (2019) *Cell Host Microbe*. | 427 |
| Dataset #11 | PRJNA495111 | Healthy | Breast milk | China | Wan Y, et al. (2020) *Gut Microbes*. | 367 |
| Dataset #12 | PRJEB34421 | Healthy | Breast milk | Spain | Boix-Amorós A, et al. (2020) *Sci Rep*. | 44 |
| **Total sample size of breast milk microbiome** | | | | **2115 samples** | | |
